# Cost-effectiveness of empagliflozin in the treatment of Malaysian patients with chronic heart failure and preserved or mildly reduced ejection fraction

**DOI:** 10.1371/journal.pone.0305257

**Published:** 2024-08-23

**Authors:** Yi Jing Tan, Stephan Linden, Siew Chin Ong

**Affiliations:** 1 Discipline of Social and Administrative Pharmacy, School of Pharmaceutical Sciences, Universiti Sains Malaysia, Penang, Malaysia; 2 Hospital Seri Manjung, Ministry of Health Malaysia, Seri Manjung, Perak, Malaysia; 3 Boehringer Ingelheim International GmbH, Ingelheim am Rhein, Germany; University of Huddersfield, UNITED KINGDOM

## Abstract

**Introduction:**

Empagliflozin demonstrates promising clinical benefits in patients with heart failure (HF). While an early study demonstrates that empagliflozin is cost-effective for treating HF patients with reduced ejection fraction (HFrEF) in Malaysia, its cost-effectiveness for HF with ejection fraction (EF)>40% remains unclear. Therefore, the current study aimed to assess the cost-effectiveness of adding empagliflozin to the standard of care (SoC) for HF patients with EF>40% from the perspective of Malaysian healthcare system. Subsequently, the results were consolidated with the findings for HFrEF to evaluate the cost-effectiveness of empagliflozin when used for all HF patients in Malaysia, irrespective of EF.

**Methods:**

A cost-utility analysis was performed using a validated Markov model, which modelled a cohort of adult patients through health states related to symptom severity and functional impairment, to estimate costs and quality-adjusted life-years (QALYs). The influence of model inputs and assumptions, sensitivity, scenario, and subgroup analyses were explored. All costs were expressed in 2022 Malaysian ringgits (RM). Costs and QALYs were discounted at an annual rate of 3.0% as per local pharmacoeconomic guideline.

**Results:**

The base-case incremental cost-effectiveness ratio (ICER) for HF patients with EF>40% was RM 40,454 per QALY gained. At a cost-effectiveness threshold of RM 47,439/QALY gained, empagliflozin was cost-effective in 57% of replications. The model outcomes were sensitive to inputs related to the treatment effect of empagliflozin in reducing HF-related hospitalisation and cardiovascular mortality, and empagliflozin cost. For the overall HF population, the ICER was RM 29,463/QALY gained.

**Conclusion:**

The findings suggest that empagliflozin is a cost-effective treatment option for the Malaysian HF population, including those with EF>40%. As such, the intervention warrants consideration by the Malaysian healthcare provider to mitigate the burden of HF and address the unmet needs of the EF>40% population.

## Introduction

Heart failure (HF) is a major public health issue affecting over 64.3 million people worldwide in 2017 [1] and its prevalence is expected to rise steadily owing to ageing populations. Prevalence estimates for HF in the general adult population currently stand between 1 and 3%, and higher at 10% in the elderly population, with notable regional differences. Although data on HF incidence are scarce both globally and locally, it is generally assumed that the incidence rate of HF ranges between 1–20 cases per 1,000 patient-years [2–4]. In Malaysia, the burden of HF is on an upward trend. According to the Global Burden of Disease Study, the age-standardised prevalence rate in 2019 was 711.9 per 100,000 individuals worldwide and 809.5 per 100,000 individuals in Malaysia. While there was a 29.4% increase in the number of HF cases worldwide between 2010 and 2019, Malaysia experienced a higher increase of 42.0% during the same period [5]. The Asian Sudden Cardiac Death in Heart Failure (ASIAN-HF) explored the characteristics of 5,276 Asian patients (including 541 Malaysians) with heart failure with reduced ejection (HFrEF) and showed that the mean age of Malaysians with HFrEF was 57.4 years old, at least a decade younger than their counterparts in the Western world [6]. The Malaysian Heart Failure (MYHF) registry, a registry-based observational study with 2,717 HF patients across the country of three ejection fraction (EF) subgroups, i.e., HFrEF, HF with mildly reduced ejection fraction (HFmrEF), and HF with preserved ejection (HFpEF), corroborated this finding by reporting a mean age of 60 years for the overall HF population, with the average age of HFrEF, HFmrEF and HFpEF patients being 57.3 years, 63.3 years, and 65.3 years, respectively. The registry data also showed that HFrEF accounted for two-thirds of HF cases whereas HFmrEF and HFpEF made up 11% and 22%, respectively [7].

People living with HF generally have a reduced life expectancy and quality of life, as well as increased needs for healthcare, thereby putting a profound strain on the local healthcare system. The economic burden of HF is enormous as HF patients frequently require hospital admissions, which have been shown to be the primary cost driver of healthcare expenditure on HF [8, 9]. A younger HF population in Malaysia aggravates the adversity even further as many would be forced to retire early, resulting in substantial losses of workforce and productivity. The indirect costs of HF, i.e., costs associated with productivity loss, were estimated to account for 94% of the 2012 estimate of total HF cost, at RM 785 million, in Malaysia [10].

Pharmacotherapy aims to alleviate symptoms and reduce hospitalisation due to HF (hHF), as well as improve quality of life and survival of HF patients. For HFrEF patients, these treatment goals can be achieved by using a combination of diuretics, renin-angiotensin system inhibitors (RASi), beta-blocker, and mineralocorticoid receptor antagonist (MRA)–known collectively as the standard of care (SoC). These treatments, however, do not confer benefits to the same extent for HFmrEF and HFpEF (or EF>40% subgroup), for whom the focus of medical therapy has so far been on symptomatic relief and optimising treatments for comorbidities. Empagliflozin (EPG), a sodium-glucose co-transporter-2 inhibitor (SGLT2i), represents a promising treatment to address this unmet need in HF management. EPG has been shown in landmark trials to significantly reduce the composite outcome of cardiovascular (CV) death or hHF in both HFrEF [hazard ratio (HR) 0.75; 95% CI 0.65–0.86] and EF>40% (HR 0.79; 95% CI 0.69–0.90) phenotypes. This observed benefit was independent of diabetes status and driven mainly by the reduction of hHF risk among EPG users [11, 12]. EPG also slowed decline in glomerular filtration rate and improved health-related quality of life (HRQoL) in both HF phenotypes [13, 14] without increasing the risk for hypotension and volume depletion [15]. Consequently, the American College of Cardiology/American Heart Association (ACC/AHA) considers EPG to be a reasonable treatment for all HF patients [16].

Adding EPG to SoC is desirable for both patients and clinicians. While its widespread adoption may increase health expenditure in the short term, it has the potential to decrease other healthcare spending in the long run. Malaysia operates a dual-tier healthcare system, consisting of public and private sectors. The public healthcare system, managed by the Ministry of Health (MOH) caters for over 70% of the population, making the MOH the country’s primary healthcare payer [17]. Due to limited healthcare resources and the potential for high utilisation of EPG, cost-effectiveness analyses serve an important tool to support informed decision making within the local context. A model-based economic evaluation has been performed to assess the cost-effectiveness of EPG as an add-on therapy to SoC versus SoC alone for the treatment of HFrEF patients in Malaysia, with a starting age of 60 years old to reflect the average age of local HF population. The analysis revealed that treating HFrEF patients with EPG is cost-effective from the perspective of the country’s primary healthcare payer, i.e., Ministry of Health (MOH), Malaysia [18]. As EF>40% represents a disparate phenotype from HFrEF, such finding may not be generalisable, and therefore calls for a separate economic evaluation for the EF>40% subgroup. Furthermore, it would also be of interest to decision makers to know whether granting all HF patients access to EPG, regardless of their EF, would be cost-effective, especially as restricting this new intervention to only a selected group, e.g., the HFrEF phenotype, may be impractical. The objectives of this study were, therefore, to perform a cost-utility analysis (CUA) of add-on EPG to SoC versus SoC alone in EF>40% patients from the perspective of Malaysian healthcare system, and to analyse the economic value of EPG when it is prescribed for HF patients irrespective of their ejection fraction by taking into consideration the findings of the early analysis for HFrEF.

## Materials and methods

### Decision model

A Markov cohort state-transition model previously developed and validated for simulating disease progression of HF patients was adapted in this study to evaluate the cost-effectiveness of EPG plus SoC versus SoC alone in HF patients with EF>40% from the perspective of Malaysian healthcare system [19, 20]. The model comprised four “alive” health states based on the Kansas City Cardiomyopathy Questionnaire (KCCQ) clinical summary score (CSS) quartiles and death. The four quartiles were as follows, KCCQ-CSS quartile 1 (0 to <55.73), KCCQ-CSS quartile 2 (55.73 to <73.96), KCCQ-CSS quartile 3 (73.96 to <88.02) and KCCS-CSS quartile 4 (88.02 to 100)–based on the distribution of patients recruited for EMPEROR-Preserved, where lower KCCQ-CSS scores represent poorer health status, more severe HF symptoms and higher levels of functional impairment [21]. KCCQ-CSS is a subscale of KCCQ that encompasses symptoms and functional impairment associated with HF and has good sensitivity to changes in health status, thus was selected as the basis for the health states in the model. This modelling approach was accepted by health technology assessment (HTA) bodies [22, 23]. The face validity and technical validity of the current model were conducted with local clinical experts and a modelling expert who ensured the model used clinically plausible inputs and was free of inconsistencies. The predictive validity was also assessed by reproducing clinical event rates and hazard ratios between treatment arms over the EMPEROR-Preserved trial duration.

The modelled cohort in the base-case analysis reflects the intention-to-treat (ITT) population of EMPEROR-Preserved trial [12]. All patients were adults (aged ≥18 years) who had symptomatic HF (NYHA class II to IV) with EF >40%, and an estimated glomerular filtration rate (eGFR) ≥20ml/min/1.73 m^2^. Of note, the trial ITT population had a mean age of 71.9 years, and this was lowered to 64.6 years as the starting age of the modelled population in the base-case analysis to reflect the mean age of local HF patients with EF>40%, as observed in the Malaysian Heart Failure (MYHF) registry. A scenario analysis in which the modelled cohort had a starting age of 71.9 years, as well a subgroup analysis in which patients with and without Type 2 diabetes (T2D) at both starting ages were evaluated separately. The mean baseline characteristics and HF treatment utilisation of the modelled populations at model entry were summarised in **Table A in S1 File**.

**Fig 1:**
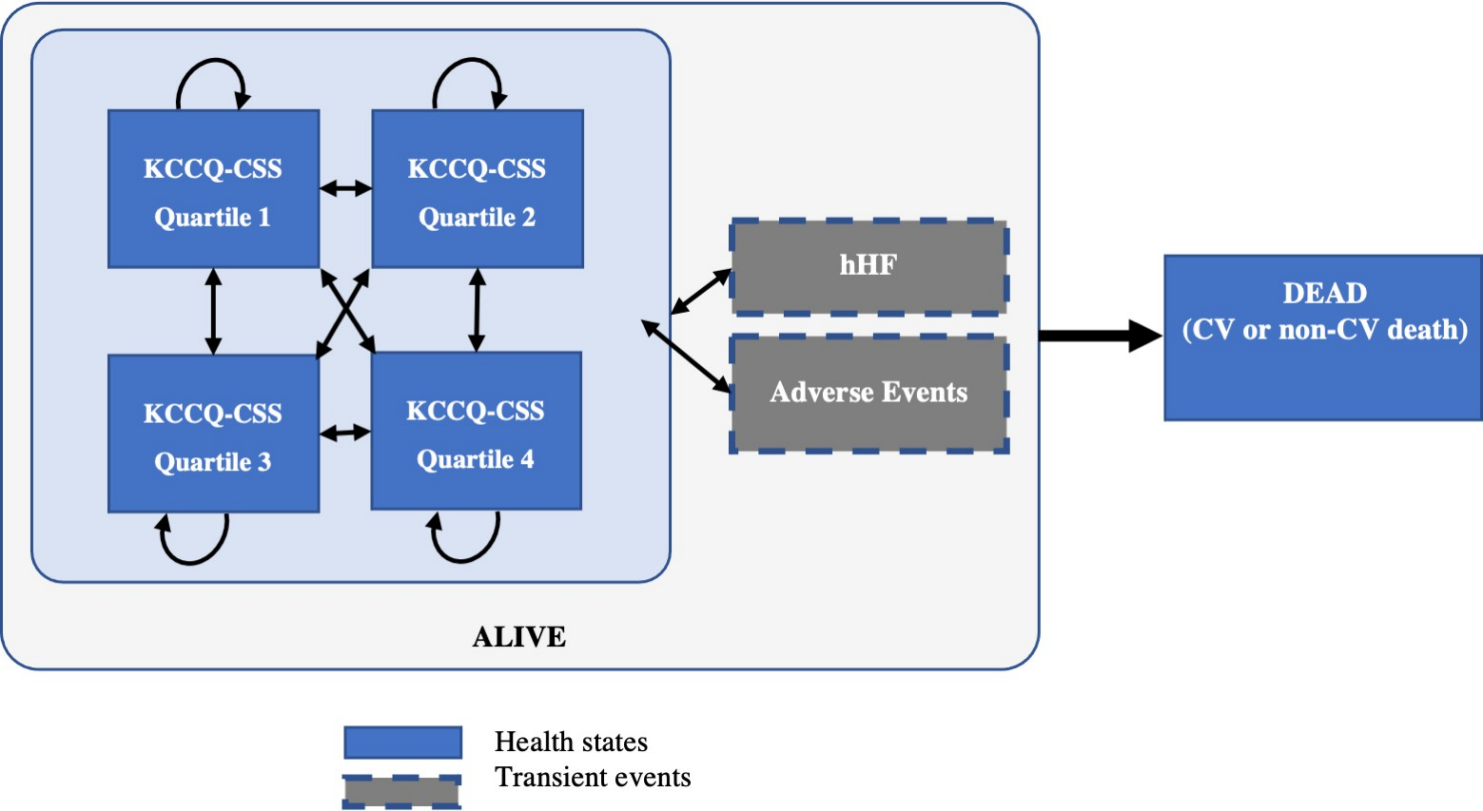
The model structure. CV = cardiovascular; hHF = hospitalisation due to heart failure; KCCQ-CSS = Kansas City Cardiomyopathy Questionnaire clinical summary score.

Both treatment arms, EPG plus SoC and SoC alone, shared the same baseline health-state distribution where approximately 25% of the cohort were in each of the KCCQ-CSS quartile health states. A lifetime horizon with a monthly cycle was chosen for the base case of the analysis and shorter time horizons were explored in scenario analyses. In each cycle, individuals could transition between KCCQ-CSS health state or die. While in any of the KCCQ-CSS health states, individuals could experience transient events such as hHF and adverse events (AEs), as illustrated in **Fig 1**. For individuals in the EPG plus SoC arm, the model also captured the proportion of individuals who discontinued EPG, who would then receive SoC only thereafter until death or the end of the modelled time horizon, wherein they experienced the same risk of clinical events as patients in the SoC arm. A scenario analysis where there was no treatment discontinuation was also carried out. **Table 1** provides a summary of base-case inputs.

**Table 1:** Base-case model inputs and sources.

Model input	Input value	Source
**Discount rate**		
Cost	3%	[24]
Benefit	3%	[24]
**Distribution for clinical parameters**		
hHF	Poisson	EMPEROR-Preserved
Time to CV death	Weibull	EMPEROR-Preserved
Time to all-cause death	Weibull	EMPEROR-Preserved
Time to EPG treatment discontinuation	Weibull	EMPEROR-Preserved
**Utility parameters**		
*Health state utility*		
KCCQ-CSS 1st quartile	0.613	EMPEROR-Preserved
KCCQ-CSS 2nd quartile	0.707	EMPEROR-Preserved
KCCQ-CSS 3rd quartile	0.778	EMPEROR-Preserved
KCCQ-CSS 4th quartile	0.832	EMPEROR-Preserved
*Disutility*		
hHF	-0.335	EMPEROR-Preserved
Urinary tract infection	-0.040	EMPEROR-Preserved
Genital infection	-0.038	[25]
Acute renal failure	-0.013	EMPEROR Preserved
Hepatic injury	-0.042	EMPEROR Preserved
Volume depletion	-0.026	EMPEROR Preserved
Hypotension	-0.025	[26]–same disutility as essential hypertension was assumed
Hypoglycaemic event	-0.002	[27, 28]
Bone fracture	-0.156	EMPEROR-Preserved
**Cost parameter**		
*Monthly drug acquisition cost*		
Empagliflozin + SoC	RM 212	Q2/2022 IQVIA pricing database
SoC	RM 101	Q2/2022 IQVIA pricing database
*Event management cost*		
hHF	RM 5,276	[9]
CV death	RM 2,573	[29] and MY-DRG Casemix Database
Non-CV death	RM 0	Assumption
*Adverse event management cost*		
Urinary tract infection	RM 213	MY-DRG Casemix Database
Genital infection	RM 320	MY-DRG Casemix Database
Acute renal failure	RM 3,185	MY-DRG Casemix Database
Hepatic injury	RM 3,230	MY-DRG Casemix Database
Volume depletion	RM 984	MY-DRG Casemix Database
Hypotension	RM 1,282	MY-DRG Casemix Database
Hypoglycaemic event	RM 693	MY-DRG Casemix Database
Bone fracture	RM 3,523	MY-DRG Casemix Database
*Disease management cost*		
KCCQ-CSS 1st quartile	RM 28	[9]
KCCQ-CSS 2nd quartile	RM 28	[9]
KCCQ-CSS 3rd quartile	RM 28	[9]
KCCQ-CSS 4th quartile	RM 28	[9]

CV = cardiovascular; EPG = empagliflozin; hHF: hospitalisation for heart failure; KCCQ-CSS = Kansas City Cardiomyopathy Questionnaire Clinical Symptom Score; SoC = standard of care

The primary outcomes of interest included life-years (LYs), quality-adjusted life years (QALYs), and costs, which were half-cycle corrected. The incremental cost-effectiveness ratio (ICER) was calculated based on the incremental costs and QALYs between the two arms. The cumulative number of clinical events experienced and event rates per 100 person-years (PY) were also reported. Future costs and benefits were discounted at annual rates of 3%, in line with the Pharmacoeconomic Guideline for Malaysia [24]. A cost-effectiveness threshold (CET) at RM 47,439 per QALY gained was used, which represents Malaysia’s gross domestic product (GDP) per capita in 2021, an approach that was considered acceptable by the local HTA agency [30]. The addition of EPG to SoC for the treatment of HF patients with EF >40% would be considered cost-effective if the ICER fell below the CET. **Table B in S1 File** provides an overview of the model characteristics. This study was exempt from institutional review board approval and patient informed consent as it only involved secondary analysis of de-identified data. The reporting of this study adhered to the 2022 Consolidated Health Economic Evaluation Reporting Standards (CHEERS) reporting guideline [31].

### Event probabilities

Clinical inputs related to efficacy of EPG treatment were derived from statistical analyses of data from the EMPEROR-Preserved trial. These include: KCCQ-CSS quartile health state transition probabilities, incidence of hHF, risk of CV death, risk of all-cause death, incidence of AEs, treatment discontinuation and health state utility values [12].

#### Transition probability matrices

The transition probabilities KCCQ-CSS quartile-defined health states for each treatment arm were determined based on longitudinal analysis of KCCQ-CSS data collected during trial, at baseline, at weeks 12, 32 and 52. It was observed that transition probabilities varied over three time periods, with inflection points at week 12 and week 32. Accordingly, three independent sets of treatment-specific transition probability matrices were derived for months 1–3, months 4–8 and months 9+ (**Table C in S2 File**). The monthly transition probabilities for patients who survived beyond 9 months were assumed to be constant until death or the end of the modelled time horizon. It was also assumed that T2D and non-T2D subgroups followed the same transition probabilities.

#### Hospitalisation for heart failure (hHF)

A repeated-measures Poisson generalised estimating equations (GEE) model was fitted to trial data to estimate the monthly rate of hHF. The risk equation included treatment effect and time-varying KCCQ-CSS quartile health state as covariates (**Table D in S2 File**).

#### Mortality

Standard parametric survival models (exponential, Weilbull, lognormal, log-logistic, Gompertz and generalised gamma) were fitted to EMPEROR-Preserved dataset, and the best-fitting distribution was selected based on visual assessment, goodness of fit criteria and clinical plausibility. The Weibull distribution was assessed to be the best distribution and thus used in the base-case analysis for estimating mortality (CV and all-cause) over time during and beyond the trial duration, adjusting for treatment allocation and time-varying KCCQ-CSS health state. Taking into account clinical plausibility as well as an observed trend towards lower CV death with EPG treatment, EPG was assumed to have a treatment effect on CV death and no effect on all-cause death. The sensitivity of the model outputs to the choice of survival distribution and the alternative assumption of no CV survival benefit from EPG treatment was assessed using deterministic sensitivity analyses. The risk of non-CV death during each cycle was estimated from the difference between the parametric risk equations for all-cause and CV death. Non-CV mortality rates based on the difference between age- and sex-specific rates of all-cause death and CV death were also calculated by using data from Malaysia’s 2020 Life Tables and Malaysian Diagnosis-Related Group (MY-DRG) Case-mix Database (where CV death was defined by ICD-10 diagnostic codes: I00-I99). In any cycle, if the trial-based estimate of non-CV death was lower than the non-CV death for the general Malaysian population, the latter was used. Such adjustment is necessary to ensure that the risk of non-CV death for HF patients should be at least as high as that observed in the general population. Details of the population- and distribution-specific risk equations for both all-cause and CV death are provided in **Tables E-H in S2 File.**

#### Treatment discontinuation

A similar approach to estimating CV mortality was adopted to estimate EPG discontinuation over time during and beyond the trial duration. The generalised gamma distribution was the best fitting distribution and used in the base case analysis, with time-varying KCCQ-CSS health state as covariates. Subgroups based on T2D status were assumed to follow the distribution. Alternative distributions for treatment discontinuation and the scenario of no treatment discontinuation were also explored in sensitivity analyses. The risk equations are provided in **Tables I and J in S2 File**. Cessation of EPG treatment in the EPG plus SoC arm was assumed to result in event risks, costs, and utilities as for SoC alone.

#### Treatment-emergent adverse events

Adverse events experienced by patients in the EMPEROR-Preserved trial with event rates of >1% (or 1 per 100-patient years) reported in either one or both treatment arms were modelled (**Table K in S2 File**). These included urinary tract infection, genital infection, acute renal failure, hepatic injury, volume depletion, hypotension, hypoglycaemic event, and bone fracture. All modelled cohorts were assumed to have the same ongoing risk of experiencing each AE. Ketoacidosis, with an event rate <1%, was excluded by default in the base-case analysis and its impact on model outputs was explored in a scenario analysis.

### Resource use and costs

In view of the perspective used in this analysis, only direct medical costs were considered in the model, which included costs associated with drug acquisition, clinical events, and disease management, sourced from local databases and published literature. All costs were expressed in 2022 Malaysian Ringgits (RM), with adjustments for inflation using the health care component of the Consumer Price Index (**Table L in S3 File**) from the Department of Statistics, Malaysia (2022) [32].

#### Drug acquisition

For the base-case analysis, the Q2/2022 IQVIA drug pricing database was used to determine the unit costs of empagliflozin 10 mg, SoC therapies, and treatments necessary for adverse events. The SoC for both arms were assumed to be equivalent and comprised of drug classes such as angiotensin-converting enzyme inhibitor (ACEi), angiotensin receptor blocker (ARB), angiotensin receptor neprilysin inhibitor (ARNi), beta-blocker (BB), mineralocorticoid receptor antagonist (MRA), and loop diuretics. To ensure relevance to local treatment practice, the member drugs were identified for each HF drug class, and their market share and daily dosages determined based on the availability in local formulary and target doses recommended by guidelines, with confirmation from cardiologists whose expert opinion was elicited using a data collection form. The monthly drug cost of SoC was then calculated as a weighted average cost based on the utilisation rates reported in EMPEROR-Preserved trial for each population. The details of drug classes, their member drugs, dosages, market share, and calculation of monthly costs of both treatment regimens (EPG plus SoC and SoC alone) are shown in **Tables M and N in S3 File**. Drug prices based on the 2022 procurement pricing from Ministry of Health Malaysia were used in a scenario analysis but were not revealed in this report due to commercial sensitivities. The current model did not include the costs of heart devices or implantation, and assumed patients on these devices already have them implanted at model entry.

#### Clinical events

The average cost of hHF per admission was sourced from a local cost-analysis study by Ong et al. (2022), which retrospectively estimated healthcare resource utilisation (HRU) and costs incurred by HF patients during a year following index hospitalisation by using a prevalence-based bottom-up, micro-costing approach [9]. The hHF cost was assumed to be equivalent for all the modelled populations (**Table 1**).

For cardiovascular (CV) mortality, which encompassed fatal coronary, cerebrovascular and HF events, the gender-specific cost of each fatal event was first estimated using a cost calculator provided by Clarke *et al*. (2010), which was designed for the estimation of costs of cardiovascular deaths for subjects of different nationalities (including Malaysians) in the Action in Diabetes and Vascular Disease (ADVANCE) trial [29]. The population-specific percentages of both male and female reported by EMPEROR-Preserved trial, the number of deaths for each type of fatal event in 2020 from Malaysian Diagnosis-Related Group (MY-DRG) Casemix Database were used to calculate the weighted average cost of CV death for the model. Details of how the weighted average cost of CV death was derived for each population is provided in **Tables O-Q in S3 File**. Non-CV deaths were assumed to incur no cost.

Treatment-emergent adverse events (AE) could be managed either in an outpatient or inpatient setting. The inpatient costs of each adverse event were computed as weighted average costs based on the reference costs and number of cases of the relevant DRG codes in the MY-DRG Casemix Database. The HRU for outpatient management of adverse events was estimated in consultation with cardiologists using a data collection form, and was used to estimate the outpatient costs of adverse events per episode alongside unit costs listed in schedules I-VII of Fees (Medical) (Cost of Services) Order 2014 [33]. Finally, the distribution of visit types (inpatient or outpatient) for AEs was elicited from clinical experts and used to calculate the weighted average cost for each AE (**Table R in S3 File**).

#### Disease management

The HRU and costs associated with outpatient HF care, including HF clinic visits and diagnostic tests, as reported by Ong et al. (2022), were used to estimate the monthly frequency and unit cost of HF care for the modelled cohort [9]. The costs of medication were excluded to avoid double counting. The monthly cost for disease management was assumed to be equivalent across HF phenotypes and KCCQ-CSS quartile health states (**Table S in S3 File)**.

### Utilities

Due to a paucity of health state utility values (HSUVs) for local HF population, most of the utility and disutility inputs were sourced from the pre-analysed EQ-5D-5L data collected in EMPEROR-Preserved trial. In the analysis, EQ-5D-5L responses were mapped to EQ-5D-3L and converted using UK value sets to utility values, to which a linear mixed-effects regression model was then fitted. The resultant equation (**Table T in S4 File**) was used to estimate utility values from individual’s baseline demographics and medical history, time-varying KCCQ-CSS health state as well as clinical events. The impacts of hHF and AEs on HRQoL were captured as one-off utility decrements, over a year for hHF and a month for AEs, to the proportion of patients who experienced these events. Disutility estimates for hypotension, genital mycotic infection, hypoglycaemia, and ketoacidosis were obtained from published literature [25–28] as they were deemed more clinically plausible than values given by the regression model (**Table 1**).

### Sensitivity analyses

One-way deterministic sensitivity analyses (DSA) were conducted to assess the uncertainty across model parameters (EPG treatment effect on event risks, EPG discontinuation, various cost categories, utility, and disutility values). Parameters were varied across plausible ranges or using alternative values as model inputs to test the robustness of model results to changes in these parameters. Subgroup and scenario analyses were also run, as described in previous sections. A probabilistic sensitivity analysis (PSA) was performed using Monte Carlo simulation, in which model inputs were varied simultaneously using probabilistic distributions, to evaluate model uncertainty (**Table U in S5 File**). To ensure a stable mean ICER was obtained, 1,000 iterations were run in the analysis. Results of sensitivity analyses were tabulated and presented graphically as appropriate.

### Combining results for HFrEF and EF>40% phenotypes

The incremental outcomes and ICER for the overall HF population were computed as weighted averages of the base-case results for EF>40% (from this study) and HFrEF from an earlier study, with all costs adjusted to 2022 values (data in file; publication in progress; the results are presented in **Table V in S5 File**), by applying a weighting factor of 0.67 according to each phenotype’s local prevalence. The MYHF registry has reported these values as 67% for HFrEF and 33% for EF>40%. Likewise, the PSA results for the combined HF population were derived by weighting the results of each simulation, where 670 and 330 sets of outcome values (costs, LYs and QALYs for both treatment arms) were randomly selected for HFrEF and EF>40%, phenotypes, respectively, and then put together into a final dataset of 1,000 sets of values to permit the calculation of a mean ICER for the overall HF population.

## Results

### Base-case analysis

Patients who were treated with EPG had lower rates of hHF (7.3 vs. 8.4 per 100 patient-years) and CV death (4.8 versus 5.1 per 100 patients-years) when compared to patients who were on SoC alone. EPG treatment led to increased incidence of adverse events, particularly urinary tract infection, genital mycotic infection, volume depletion and hypotension, but lower rates of acute renal failure, hepatic injury, and hypoglycaemic events. Patients who received EPG were on the treatment for 3.34 years on average before discontinuation. EPG increased LYs by 0.07 and QALYs by 0.10 (**Table 2**), and total lifetime costs by RM 3,941 (**Table 3**). The cost per QALY gained with add-on EPG was RM 40,454 (**Table 4**), below the cost-effectiveness threshold of RM 47,439 per QALY gained.

**Table 2:** Summary of discounted clinical outcomes for the base-case analysis.

Result	EPG + SoC	SoC	Incremental
	Event rates (per 100 patient-years)		
hHF	7.31	8.45	-1.14
CV death	4.84	5.12	-0.28
Non-CV death (adjusted)	11.17	11.11	0.05
Adverse event (aggregate)	30.62	28.91	1.71
Urinary tract infection	5.08	4.53	0.55
Genital mycotic infection	0.82	0.39	0.43
Acute renal failure	7.05	7.26	-0.21
Hepatic injury	2.43	2.84	-0.41
Volume depletion	6.13	5.38	0.75
Hypotension	5.38	4.80	0.58
Hypoglycaemic event	1.36	1.41	-0.05
Bone fracture	2.37	2.30	0.07
**Time on EPG (undiscounted), LYs and QALYs (discounted) per patient**			
**Time receiving EPG (years)**	3.34	-	-
**Total LYs**	5.54	5.47	0.07
KCCQ-CSS 1st Quartile	1.09	1.15	-0.06
KCCQ-CSS 2nd Quartile	1.27	1.32	-0.05
KCCQ-CSS 3rd Quartile	1.33	1.28	0.05
KCCQ-CSS 4th Quartile	1.86	1.72	0.14
**Total QALYs**	4.00	3.91	0.10
KCCQ-CSS 1st Quartile	0.67	0.70	-0.04
KCCQ-CSS 2nd Quartile	0.89	0.93	-0.04
KCCQ-CSS 3rd Quartile	1.04	1.00	0.04
KCCQ-CSS 4th Quartile	1.55	1.43	0.12
Loss due to hHF	-0.135	-0.155	0.02
Loss due to AEs	-0.005	-0.005	0.00

AE = adverse event; CV = cardiovascular; EPG = empagliflozin; hHF: hospitalisation for heart failure; LY = life year; KCCQ-CSS = Kansas City Cardiomyopathy Questionnaire Clinical Symptom Score; QALY = quality-adjusted life year; SoC = standard of care

**Table 3:** Summary of discounted cost outcomes per patient for the ITT population.

Result	EPG + SoC	SoC	Incremental
	Cost outcomes (discounted), per patient		
**Drug acquisition cost**	RM10,860	RM6,629	RM4,231
**Clinical event management cost**	RM2,796	RM3,147	-RM351
hHF	RM2,123	RM2,442	-RM319
CV death	RM673	RM705	-RM32
Non-CV death	RM0	RM0	RM0
**AE management cost**	RM2,986	RM2,949	RM36
**Disease management cost**	RM1,895	RM1,870	RM25
KCCQ-CSS 1st Quartile	RM371	RM392	-RM21
KCCQ-CSS 2nd Quartile	RM433	RM450	-RM18
KCCQ-CSS 3rd Quartile	RM456	RM439	RM17
KCCQ-CSS 4th Quartile	RM635	RM588	RM47
**Total cost**	RM18,536	RM14,595	RM3,941

AE = adverse event; CV = cardiovascular; EPG = empagliflozin; hHF = hospitalisation for heart failure; KCCQ-CSS = Kansas City Cardiomyopathy Questionnaire Clinical Symptom Score; SoC = standard of care

**Table 4:** Summary of cost-effectiveness results for base-case analysis.

Outcome	EPG + SoC	SoC	Incremental
Total cost (discounted)	RM18,536	RM14,595	RM3,941
Total LYs (discounted)	5.54	5.47	0.07
Total QALYs (discounted)	4.00	3.91	0.10
**ICER, cost per LY gained**	RM54,665		
**ICER, cost per QALY gained**	RM40,454		

EPG = empagliflozin; ICER = incremental cost-effectiveness ratio; LY = life years; QALYs = quality-adjusted life years; SoC = standard of care

### One-way deterministic sensitivity analyses

**Fig 2:**
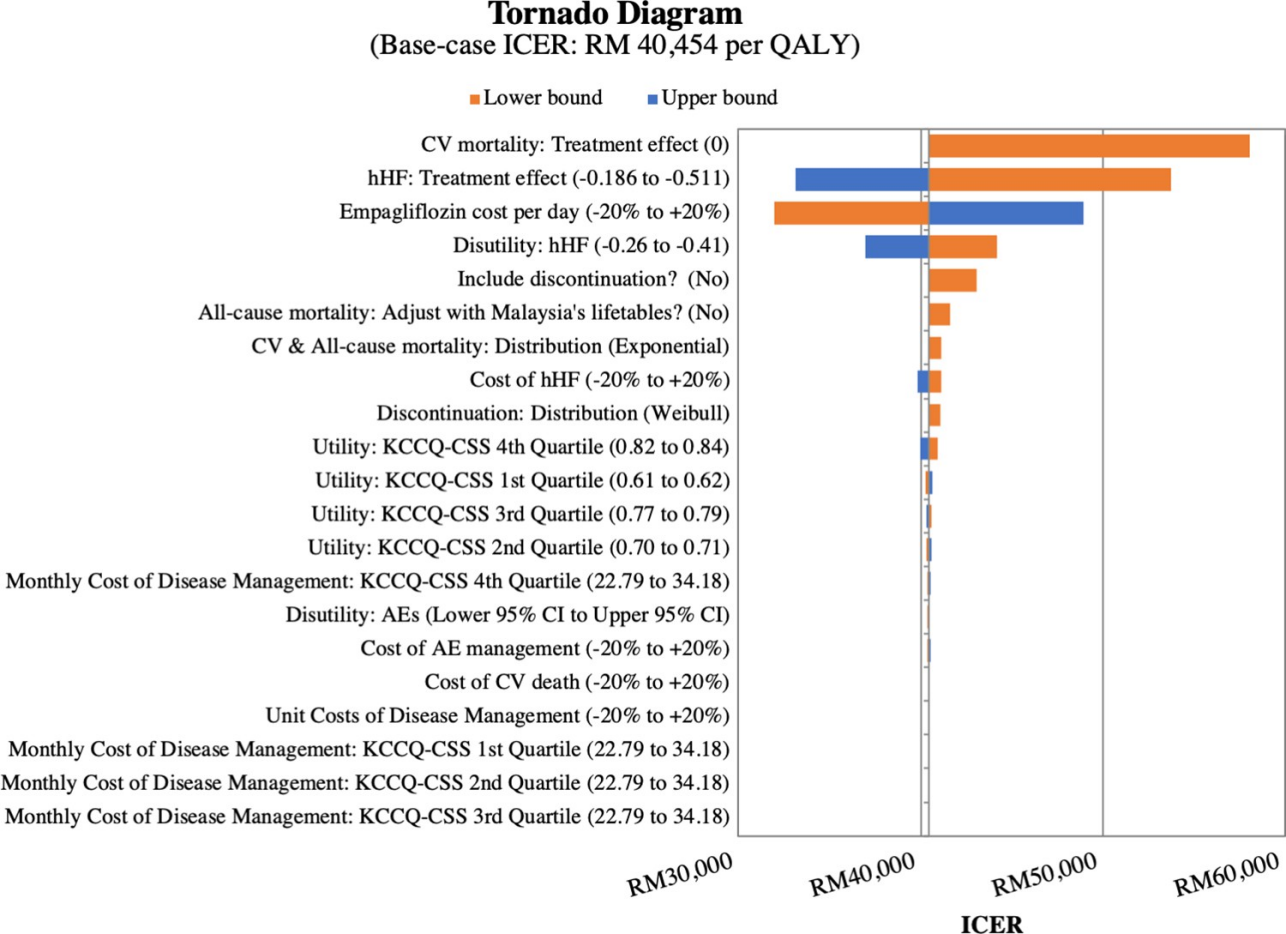
Tornado diagram demonstrating one-way sensitivity analyses for relevant parameters (for ITT population with EF>40%). AE = adverse event; CI = confidence interval; CV = cardiovascular; hHF = hospitalisation for heart failure; ICER = incremental cost-effectiveness ratio; KCCQ-CSS = Kansas City Cardiomyopathy Questionnaire Clinical Symptom Score; QALY = quality-adjusted life year.

Results of DSA are depicted by a Tornado diagram (**Fig 2**). The model was most sensitive to inputs surrounding the treatment effect of EPG on both CV death and hHF. EPG plus SoC would no longer be cost-effective compared with SoC alone if the effect of CV death reduction of EPG was removed, or when the upper limit of the 95% confidence interval (CI) for the hazard ratio (HR) of hHF was applied (HR = 0.83). Increasing EPG price by 20% also resulted in a ICER higher than the cost-effectiveness threshold of RM 47,439 per QALY gained. The ICER remained less than the threshold when other model inputs were varied over their clinically plausible ranges. Details of DSA results are given in **Table W in S6 File.**

### Probabilistic sensitivity analysis

**Fig 3:**
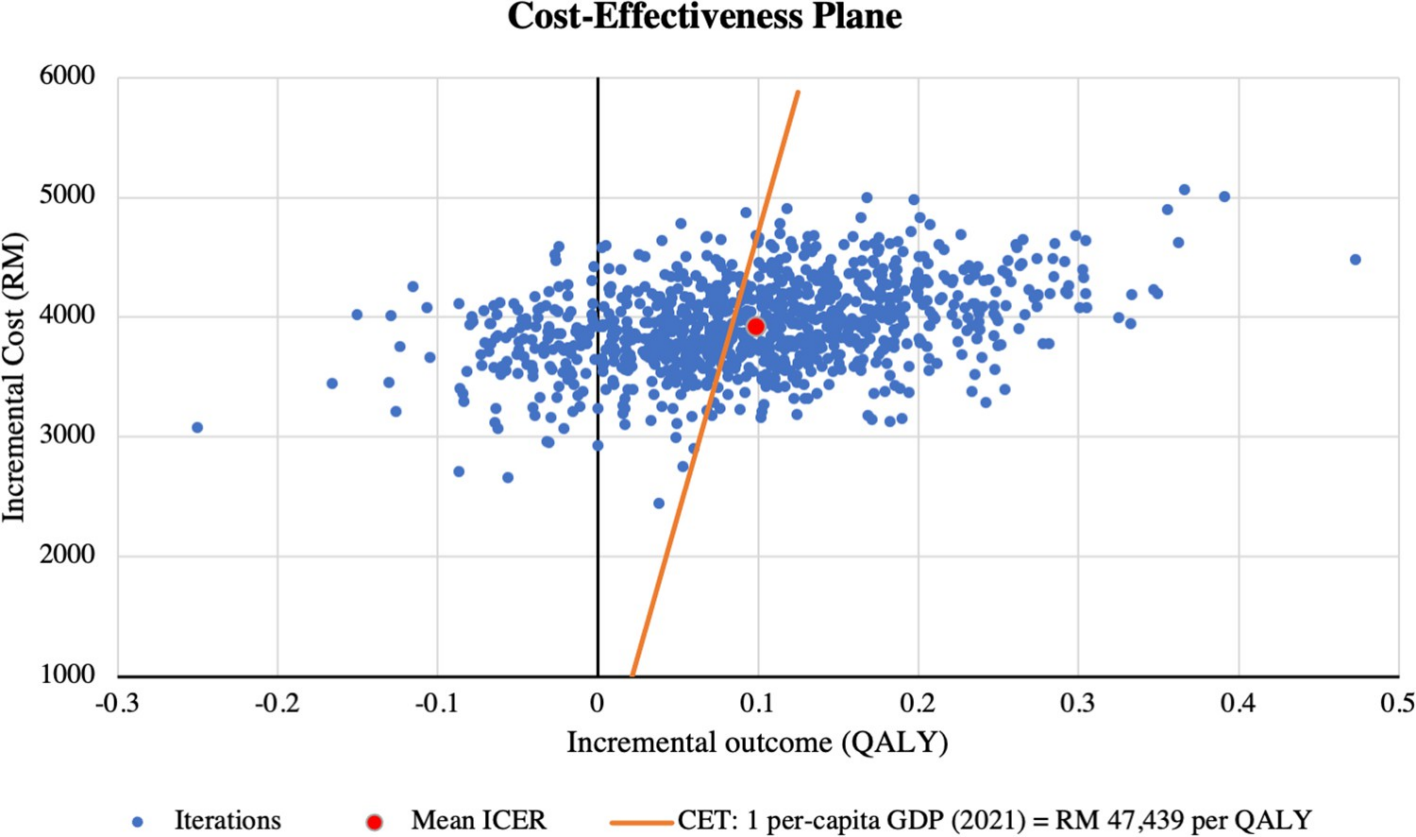
PSA scatterplot on cost-effectiveness plane (for ITT population with EF >40%). Note: The red marker represents the base-case estimate. CET = cost-effectiveness threshold; GDP = gross domestic product; ICER = incremental cost-effectiveness ratio; PSA = probabilistic sensitivity analysis; QALY = quality-adjusted life year.

**Fig 4:**
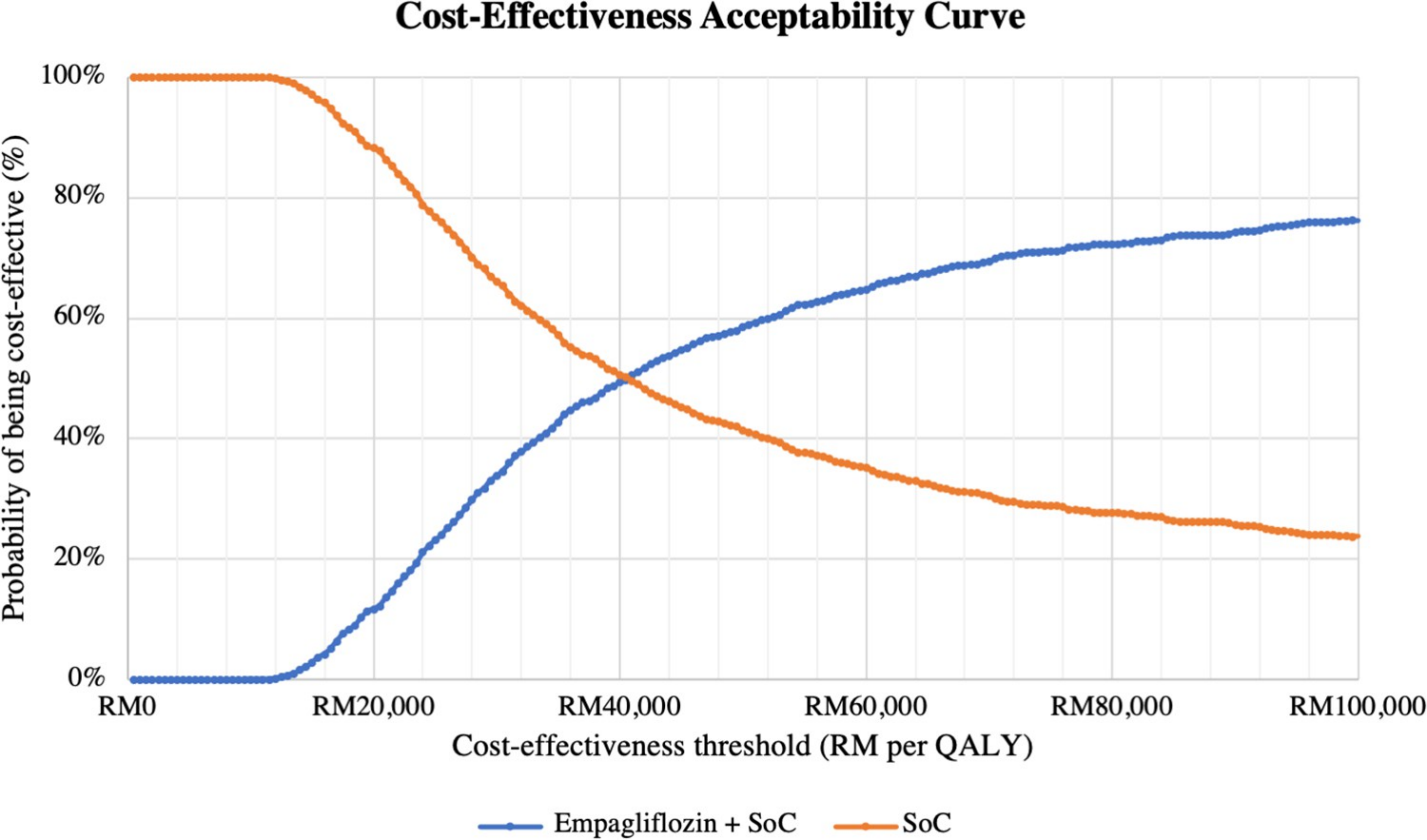
Cost-effectiveness acceptability curve comparing two treatment arms (for ITT population with EF >40%). Note: At RM 47,439 per QALY, the probabilities of EPG + SoC and SoC alone being cost effective were 57% and 43%, respectively. QALY = quality-adjusted life year; SoC = standard of care.

Results of the PSA are presented in **Table 5**. When all input parameters were simultaneously drawn from their assigned probability distributions, EPG plus SoC was cost-effective in 57% of 1,000 Monte Carlo simulations at the cost-effectiveness threshold of RM 47,439 per QALY, as shown in **Figs 3** and **4**.

**Table 5:** Outputs of probabilistic sensitivity analysis of base-case population.

Outcome	Mean	Lower 95% CI	Upper 95% CI
Incremental cost	RM3,926	RM3,190	RM4,642
Incremental QALY	0.10	-0.07	0.28
**Mean ICER, cost per QALY gained**	RM39,947		

CI = confidence interval; ICER = incremental cost-effectiveness ratio; LY = life years; QALYs = quality-adjusted life years

### Subgroup and scenario analyses

In scenario analyses (**Table 6**), there was generally little impact on ICER when a discount rate of 0% or 5% was applied and when an alternative distribution was used to extrapolate time to mortality and EPG discontinuation. However, the model was sensitive to changes in drug prices, time horizon, and the starting age of the modelled cohort. EPG plus SoC was very cost-effective when MOH procurement prices were used, with the ICER more than halved at RM16,988 per QALY gained compared to RM 40,454 per QALY gained in the base-case analysis. When the time horizon was shortened or the trial-reported mean age for the ITT population (71.9 years) was used, ICER increased drastically. The ICER was above the threshold when the modelled population consisted of only individuals who had comorbid T2D at baseline due to a smaller QALY gained (0.03) compared to the non-T2D subgroup (0.13) despite incurring a lower incremental cost (RM 1,512 vs RM 1,799). This could be attributed to the differential hazard ratios for CV mortality being used for both subgroups (HR = 1.01 for T2D subgroup and HR = 0.83 for non-T2D group), and a slightly smaller effect of EPG on hHF in the T2D subgroup than the non-T2D subgroup (HR = 0.78 versus HR = 0.77, respectively).

**Table 6:** Results of subgroup and scenario analyses.

Scenario/subgroup		ICER (RM/QALY gained)	% change from base-case ICER (RM40,454/QALY gained)
**Using Malaysian Ministry of Health (MOH) procurement pricing report (base-case: iQVIA drug pricing database)**			
1.	MOH procurement pricing database	RM16,988	-58%
**Starting age of modelled cohort (base-case: 64.6 years)**			
2.	Trial-reported mean age for ITT population: 71.9 years was used as the starting age	RM50,732	+25%
**Time horizon (base-case: Lifetime)**			
3.	5 years	RM59,271	+47%
4.	10 years	RM44,533	+10%
**Discount rate (base-case: 3%)**			
5.	0%	RM37,800	-7%
6.	5%	RM42,157	+4%
**Parametric survival model used to extrapolate CV death and all-cause death (base-case: Weilbull)**			
7.	Lognormal	RM42,679	6%
8.	Loglogistic	RM41,603	+3%
9.	Exponential	RM41,111	+2%
10.	Generalised gamma	RM39,490	-2%
11.	Gompertz	RM46,333	+15%
**Parametric survival model used to extrapolate treatment discontinuation (base-case: Generalised gamma)**			
12.	Weilbull	RM41,084	+2%
13.	Lognormal	RM41,842	+3%
14.	Loglogistic	RM41,527	+3%
15.	Exponential	RM40,886	+1%
16.	Gompertz	RM41,035	+1%
**Ketoacidosis as one of the AEs in the model (base-case: No)**			
17.	Ketoacidosis was considered as a potential AE.	RM40,288	0%
**Subgroup by T2D status**			
18.	T2D subgroup	RM57,487	+42%
19.	Non-T2D subgroup	RM31,344	-23%

AE: adverse event; CV = cardiovascular; hHF = hospitalisation for heart failure; ICER = incremental cost-effectiveness ratio; MOH = Ministry of Health (Malaysia); QALYs = quality-adjusted life years; T2D: Type 2 diabetes

### Combined analysis for overall HF population

**Fig 5:**
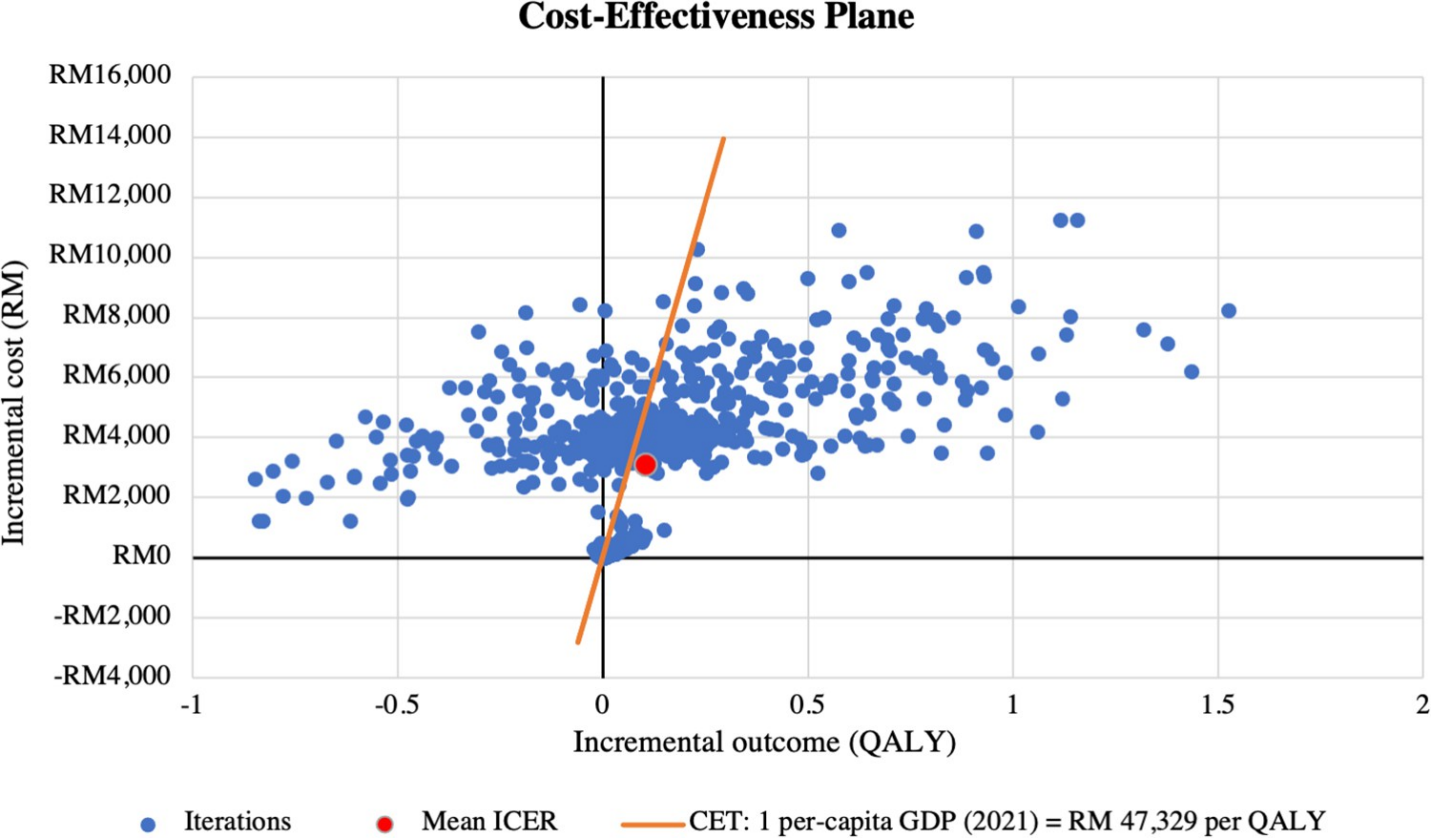
PSA scatterplot on cost-effectiveness plane (for overall HF population). Note: The red marker represents the base-case estimate. CET = cost-effectiveness threshold; GDP = gross domestic product; ICER = incremental cost-effectiveness ratio; QALY = quality-adjusted life year.

**Fig 6:**
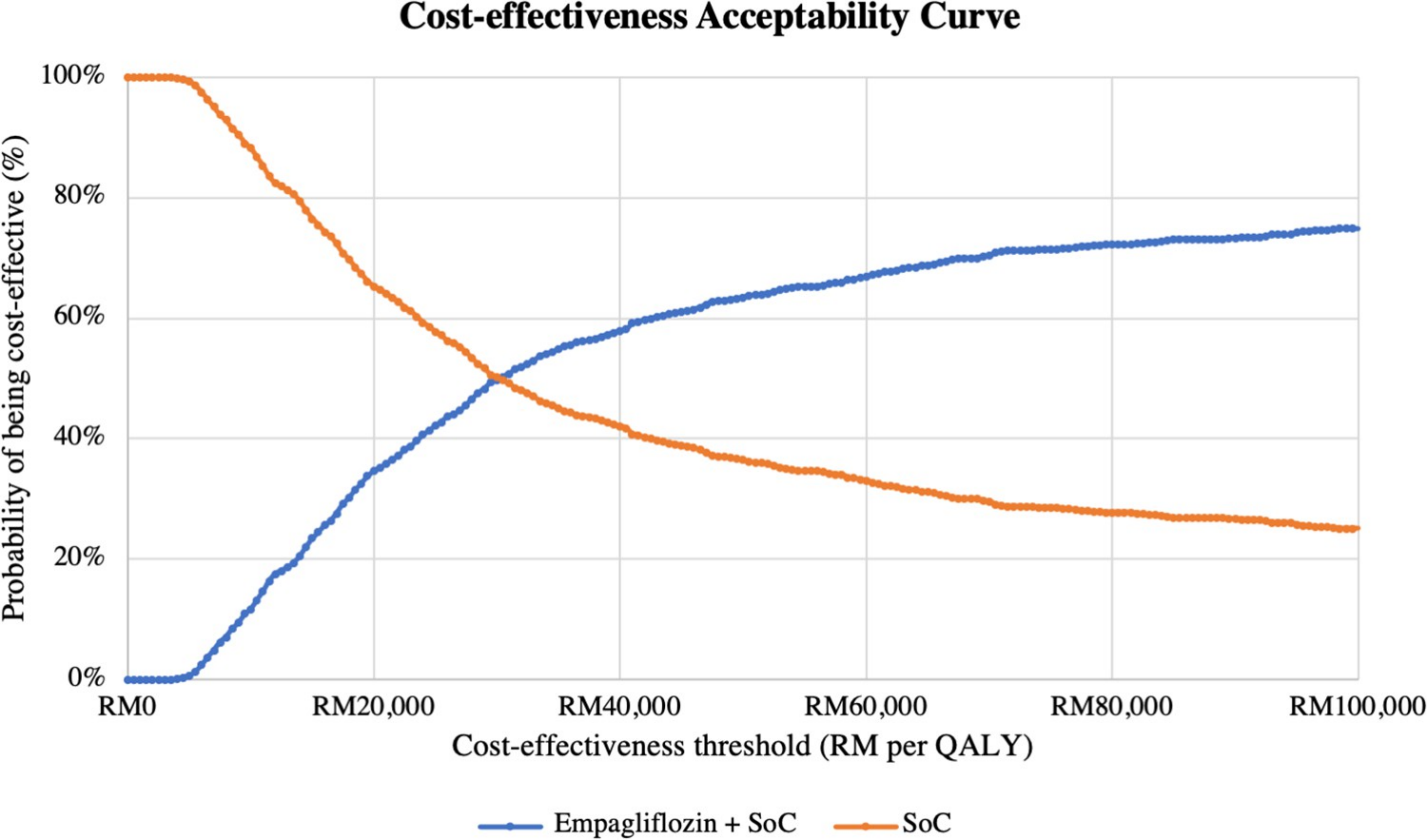
Cost-effectiveness acceptability curve for EPG plus SoC versus SoC alone (for overall HF population). Note: At RM 47,439 per QALY, the probabilities of EPG + SoC and SoC alone being cost effective were 63% and 37%, respectively. QALY = quality-adjusted life year; SoC = standard of care.

**Table 7** shows the deterministic and probabilistic cost-effectiveness outcomes of combining the results of the base-case analyses for EF>40% subgroup and HFrEF from a prior study (see **Table V in S5 File**). 62.8% of 1,000 iterations (67% randomly sampled from the PSA results for HFrEF cohort and 33% from the EF>40% cohort), were below the threshold of RM 47,439/QALY gained (**Figs 5** and **6**).

**Table 7:** Results of combined analysis for overall HF population.

Outcome	Deterministic	Probabilistic	
		Mean	95% CI
Incremental cost	RM3,747	RM 3,113	RM 36 to RM 8,028
Incremental QALY	0.15	0.11	-0.44 to 0.87
ICER, cost per QALY gained	RM24,605	RM29,463	

CI = confidence interval; ICER = incremental cost-effectiveness ratio; LY = life years; QALYs = quality-adjusted life years

## Discussion

Our base-case results showed that EPG remained a cost-effective treatment for HF patients with EF>40% from the perspective of the Malaysian healthcare system, even though the ICER obtained was twice the ICER from an earlier study for the HFrEF population (i.e., RM 40,454 per QALY gained versus RM 20,364 per QALY gained). The difference in model outcomes may be explained by a relatively lower baseline risk of hHF and CV mortality among EF>40% patients when compared to HFrEF patients. The model outcomes were robust to a series of DSA and scenario analyses that addressed the uncertainties in model structure and parameters. However, the model outcomes were sensitive to the inputs relating to the treatment effect of EPG on CV death and hHF, where EPG was no longer cost-effective when the HR was raised to 1 (for CV death) or the upper limit of the 95% CI (0.83 for hHF). Similarly, the T2D subgroup, which had less favourable HRs for these clinical outcomes, recorded higher ICERs than the ITT population and non-T2D subgroup. EPG was not cost-effective over a shorter time horizon of 5 years, or when the starting age of the modelled population was 71.9 years (since most Malaysians would die as they reach the age of 80 according to Malaysian life tables). This finding suggests that EPG treatment provides more economic benefit in the long run. Lowering EPG price or using MOH procurement prices (which were lower than IQVIA drug prices) could improve the economic value of EPG among patients with EF>40%. Combined analysis of results for both HFrEF and EF>40% population reassuringly showed that prescribing EPG for all HF patients, regardless of phenotype, was cost-effective. These finding have important implications for the country’s Ministry of Health (MOH), which is the primary provider of medical treatment for approximately two-thirds of local population, including HF patients, according to the 2019 National Health and Morbidity Survey (NHMS) [17]. MOH stakeholders could be reassured that at the current negotiated price (MOH drug pricing), adding EPG to SoC for HF patients treated in MOH, regardless of ejection fraction, would represent a cost-effective use of healthcare sources.

The findings of the current study generally agreed with other studies evaluating the cost-effectiveness of EPG in HF patients with EF>40% across various jurisdictions, including Australia, China, Finland, Thailand, and the United States with particular respect to parameters that are most impactful to ICER, including the efficacy of EPG on hHF and CV death, and the cost of EPG [20, 34–40]. In Thailand, adding EPG to SoC to treat EF>40% patients resulted in an ICER that was above the locally accepted cost-effectiveness threshold as the study did not factor in the treatment effect of EPG on CV death and reported a higher EPG price than the local setting [35]. Similar results were reported by Zheng et al. (2022) [38], who provided evidence that in the absence of CV benefit, EPG provided low economic value from the perspective of US healthcare system despite halving EPG price and using a higher CET.

Our model has several limitations that warrant considerations when interpreting the study results. Due to the lack of local data, the current CUA relied on pre-analysed data from the EMPEROR-Preserved trial to inform most model inputs, including patient characteristics, drug utilisation, health state transition probability matrices, risk equations, AE incidence rates, treatment discontinuation rates, health state utility values, and event dis-utilities. This approach has been adopted in multinational CUAs to address data limitations, aiming to provide the most accurate economic estimates possible [19, 20, 41]. Where possible, local data were incorporated, such as adjusting age and life expectancy of the modelled population to better align with local demographics, utilising local HRU and cost data, and consulting local clinical experts to confirm the face validity of our analyses. These steps were taken to further improve generalisability of our findings to the local context. As the model relied on extrapolations to predict long-term outcomes of the modelled population, there was uncertainty in the model outputs. This was addressed through a series of DSA and scenario analyses using alternative distributions, which did not affect the conclusion. However, the uncertainty surrounding the effect of EPG on CV death could affect the economic value of EPG. While meta-analyses suggest that EPG may offer modest benefits in reducing CV mortality for the overall HF population, real-world studies are essential to confirm the longer-term CV outcomes of HF patients with EF>40% receiving EPG. Owing to a paucity of local data, we relied on pre-analysed HRQoL data from the EMPEROR-Preserved trial and published literature from other jurisdictions for utility and disutility estimates. To account for the uncertainty surrounding the generalisability of these values to the local population, a range of sensitivity analyses were performed, which showed that EPG remained cost-effective when utility and disutility estimates were varied across their clinically plausible ranges. This limitation calls for future research into derivation of utility and disutility values for HF patients in Malaysia to support future economic evaluations.

## Conclusions

Results of this cost-effectiveness analysis suggest that EPG treatment for HF patients with EF >40% is cost-effective from the perspective of Malaysian healthcare system, albeit to a lesser extent compared to the HFrEF subgroup. The consolidated results for both HFrEF and EF >40% patients indicate that EPG represents a cost-effective use of health resources and should be considered for all HF patients regardless of their ejection fraction in Malaysia.

## Supporting information

S1 File Decision model. (DOCX)

S2 File Event probabilities. (DOCX)

S3 File Costs. (DOCX)

S4 File Utilities. (DOCX)

S5 File Sensitivity analyses. (DOCX)

S6 File Results. (DOCX)
