# Supplementary material for: Cost-effectiveness of empagliflozin in the treatment of Malaysian patients with chronic heart failure and preserved or mildly reduced ejection fraction
Source: PLoS One. 2024 Aug 23;19(8):e0305257. doi: 10.1371/journal.pone.0305257 (PMC11343421; doi:10.1371/journal.pone.0305257)
Supplement: S1 File — (DOCX) [file pone.0305257.s001.docx]

# S1 File 1. Decision Model

Table A. Baseline characteristics of trial ITT population and subgroups based on T2D status

| Baseline characteristic | ITT (base case analysis) | Subgroup analysis | |
| --- | --- | --- | --- |
|  |  | With T2D | Without T2D |
| **Demographics** |  |  |  |
| Age (years) | 71.9* | 70.9* | 72.8* |
| Sex: Male | 55.3% | 57.2% | 53.4% |
| **Treatment use at baseline** |  |  |  |
| ACEi | 40.2% | 40.2% | 40.3% |
| ARB | 38.7% | 41.6% | 35.8% |
| ARNi | 2.2% | 2.1% | 2.3% |
| MRA | 37.5% | 39.1% | 35.9% |
| BB | 86.3% | 87.9% | 84.8% |
| Ivabradine | 1.2% | 1.2% | 1.1% |
| **Medical history** |  |  |  |
| Ischaemic cause of heart failure | 35.4% | 41.4% | 29.5% |
| Prior atrial fibrillation or flutter | 52.4% | 46.9% | 57.6% |
| Comorbid T2D | 49.1% | 100.0% | - |
| Comorbid CKD (eGFR <60 mL/min/1.73 m^2^) | 40.9% | 51.4% | 48.5% |

ACEi = angiotensin-converting enzyme inhibitor; ARB = angiotensin receptor blocker; ARNi = angiotensin receptor neprilysin inhibitor; BB = beta blocker; CKD = chronic kidney disease; CSS = clinical summary score; eGFR = estimated glomerular filtration rate; HF = heart failure; ITT = intent to treat; KCCQ = Kansas City Cardiomyopathy Questionnaire; MRA = mineralocorticoid receptor antagonist; MYHF = Malaysia Heart Failure registry; NYHA = New York Heart Association; T2D = type 2 diabetes

*For the base case of the analysis, 64.6 years was selected as the starting age of modelled cohort in line with the average age of EF>40% patients reported by Malaysian Heart Failure (MYHF) registry (1). For scenario analyses, the reported mean ages of the trial populations were used (2).

Table B. Key model characteristics

|  | Description |
| --- | --- |
| Type of evaluation | Cost-utility analysis |
| Patient population | Patients, aged 18 years or above, with HF and LVEF >40% and in NYHA class II-IV, eGFR >20 mL/min/1.73m^2^, i.e., ITT population from EMPEROR-Preserved trial |
| Subgroup | T2D and without T2D |
| Perspective | Malaysian healthcare system |
| Time horizon | Lifetime (base case). Shorter horizons were explored in the scenario analyses. |
| Intervention | Empagliflozin + SoC |
| Comparators | Placebo + SoC, comprised of the following:   - ACEi (enalapril, perindopril, ramipril) - ARB (losartan, valsartan) - ARNi (sacubitril / valsartan) - BB (bisoprolol, carvedilol) - Ivabradine - MRA (spironolactone) - Loop diuretics (frusemide, bumetanide) |
| Model approach | Markov cohort state-transition approach, with monthly cycles, based on discrete health states characterised by KCCQ-CSS score quartiles |
| CV and death events | - hHF - CV death - Non-CV death |
| Treatment-related AEs | - Urinary tract infection - Genital mycotic infection - Acute renal failure - Hepatic injury - Volume depletion - Hypotension - Hypoglycaemic event* - Bone fracture - Ketoacidosis** |
| HRQoL | - Utility value for each KCCQ-CSS quartile - Dis-utilities for treatment-related AEs and hHF |
| Cost elements | - Drug acquisition - Clinical event management - Disease management |
| Outcomes | - Cumulative incidence of clinical events - Total cost and costs by component - Life expectancy - QALYs - Incremental outcomes - ICER |
| Cost-effectiveness threshold | RM 47,439 per QALY gained (Malaysia’s 2021 per-capita GDP) (3) |
| Uncertainty analyses | - One-way deterministic sensitivity analyses - Probabilistic sensitivity analyses - Subgroup and scenario analyses |
| Model platform | Microsoft Excel® |

ACEi = angiotensin-converting enzyme inhibitor; AE = adverse event; ARB = angiotensin receptor blocker; ARNi = angiotensin receptor-neprilysin inhibitor; BB = beta-blocker; CV = cardiovascular; GDP = gross domestic product; HF = heart failure; hHF = hospitalisation due to heart failure; HRQoL = health-related quality of life; ICER = incremental cost-effectiveness ratio; ITT = intent to treat; KCCQ-CSS = Kansas City Cardiomyopathy Questionnaire clinical summary score; LVEF = left ventricular ejection fraction; MRA = mineralocorticoid receptor antagonist; NYHA = New York Heart Association; QALY = quality-adjusted life year; SoC = standard of care; T2D = type 2 diabetes

* Defined as an event with a plasma glucose value of ≤3.9 mmol/L or where assistance was required.

** Ketoacidosis is not included in the base case, but only as option for scenario analysis

References

1. Abidin HAZ, Kader MASA, Ross NT, Ramli AW, Ghazi AM, Hassan HHC, et al. Demographic characteristics differences across EF subgroups in the Malaysian heart failure (MyHF) registry. International Journal of Cardiology. 2021 Dec 1;345:5–6.

2. Anker SD, Butler J, Filippatos G, Ferreira JP, Bocchi E, Böhm M, et al. Empagliflozin in Heart Failure with a Preserved Ejection Fraction. New England Journal of Medicine. 2021 Oct 14;385(16):1451–61.

3. Department of Statistics Malaysia. Gross Domestic Product (GDP) By State 2021 [Internet]. Department of Statistics Malaysia; 2022 [cited 2022 Oct 10]. Available from: https://www.dosm.gov.my/v1/index.php?r=column/cthemeByCat&cat=491&bul_id=L3NnMU44VnA0YjRxVXhuYUpZTmVnZz09&menu_id=TE5CRUZCblh4ZTZMODZIbmk2aWRRQT09
