# Supplementary material for: Cost-effectiveness of empagliflozin in the treatment of Malaysian patients with chronic heart failure and preserved or mildly reduced ejection fraction
Source: PLoS One. 2024 Aug 23;19(8):e0305257. doi: 10.1371/journal.pone.0305257 (PMC11343421; doi:10.1371/journal.pone.0305257)
Supplement: S2 File — (DOCX) [file pone.0305257.s002.docx]

# S2 File. Event Probabilities

Table C. Treatment-specific transition probability matrices

| KCCQ-CSS Transitions [From, To] | EPG + SoC | | | SoC | | |
| --- | --- | --- | --- | --- | --- | --- |
|  | Months 1-3 | Months 4-8 | Months 9+ | Months 1-3 | Months 4-8 | Months 9+ |
| KCCQ [1,1] | 0.814 | 0.912 | 0.926 | 0.828 | 0.913 | 0.926 |
| KCCQ [1,2] | 0.158 | 0.076 | 0.063 | 0.138 | 0.072 | 0.060 |
| KCCQ [1,3] | 0.023 | 0.010 | 0.011 | 0.033 | 0.011 | 0.010 |
| KCCQ [1,4] | 0.005 | 0.002 | 0.000 | 0.001 | 0.004 | 0.004 |
| KCCQ [2,1] | 0.067 | 0.063 | 0.064 | 0.090 | 0.064 | 0.056 |
| KCCQ [2,2] | 0.731 | 0.840 | 0.847 | 0.742 | 0.848 | 0.867 |
| KCCQ [2,3] | 0.165 | 0.081 | 0.085 | 0.140 | 0.077 | 0.066 |
| KCCQ [2,4] | 0.037 | 0.016 | 0.004 | 0.028 | 0.011 | 0.011 |
| KCCQ [3,1] | 0.017 | 0.005 | 0.003 | 0.010 | 0.005 | 0.011 |
| KCCQ [3,2] | 0.091 | 0.077 | 0.078 | 0.093 | 0.076 | 0.074 |
| KCCQ [3,3] | 0.723 | 0.838 | 0.842 | 0.735 | 0.848 | 0.845 |
| KCCQ [3,4] | 0.169 | 0.080 | 0.077 | 0.162 | 0.071 | 0.070 |
| KCCQ [4,1] | 0.005 | 0.003 | 0.000 | 0.001 | 0.004 | 0.001 |
| KCCQ [4,2] | 0.010 | 0.009 | 0.006 | 0.016 | 0.008 | 0.008 |
| KCCQ [4,3] | 0.085 | 0.058 | 0.052 | 0.090 | 0.053 | 0.056 |
| KCCQ [4,4] | 0.900 | 0.930 | 0.942 | 0.893 | 0.935 | 0.935 |

CSS = clinical summary score; EPG = empagliflozin; KCCQ = Kansas City Cardiomyopathy Questionnaire; SoC = standard of care

Table D. Population-specific risk equations for hHF (Poisson distribution)

|  | Coefficient | | | SE |
| --- | --- | --- | --- | --- |
| Covariate | **ITT population** | **T2D** | **Non-T2D** |  |
| Intercept | -4.291 | -4.134 | -4.518 | 0.284 |
| EPG treatment effect | -0.254 | -0.244 | -0.265 | 0.296 |
| Updated KCCQ-CSS: relative to KCCQ-CSS 0 to <55.73 (Quartile 1) | | | | |
| KCCQ-CSS: 55.73 to <73.96 (Quartile 2) | -0.653 | -0.552 | -0.764 | 0.297 |
| KCCQ-CSS: 73.96 to <88.02 (Quartile 3) | -1.074 | -1.063 | -1.028 | 0.306 |
| KCCQ-CSS: 88.02 to 100 (Quartile 4) | -1.160 | -1.101 | -1.170 | 0.332 |

EPG = empagliflozin; KCCQ-CSS = Kansas City Cardiomyopathy Questionnaire Clinical Symptom Score; SE = standard error; T2D = type 2 diabetes

Table E. Population-specific risk equations for all-cause death (Weibull distribution)

|  | Coefficient | | | SE |
| --- | --- | --- | --- | --- |
| Covariate | **ITT population** | **T2D** | **Non-T2D** |  |
| Shape | 0.323 | 0.329 | 0.315 | 0.177 |
| Scale | -10.716 | -10.753 | -10.677 | 0.264 |
| EPG treatment effect | 0.016* | 0.073* | -0.049 | 0.246 |
| Updated KCCQ-CSS: relative to KCCQ-CSS 0 to <55.73 (Quartile 1) | | | | |
| KCCQ-CSS: 55.73 to <73.96 (Quartile 2) | -0.332 | -0.311 | -0.344 | 0.257 |
| KCCQ-CSS: 73.96 to <88.02 (Quartile 3) | -0.601 | -0.579 | -0.609 | 0.272 |
| KCCQ-CSS: 88.02 to 100 (Quartile 4) | -0.969 | -0.863 | -1.064 | 0.279 |

EPG = empagliflozin; KCCQ-CSS = Kansas City Cardiomyopathy Questionnaire Clinical Symptom Score; SE = standard error; T2D = type 2 diabetes

* Set to zero to ensure clinical plausibility

Table F. Risk equations for all-cause death based on alternative distributions for ITT

| Covariate | Exponential | Gompertz | Log-Normal | Log-Logistic | Generalised Gamma |
| --- | --- | --- | --- | --- | --- |
| P1 | - | 0.001 | 1.505 | 1.452 | 0.536 |
| P2 (intercept) | 0.000(285) | 0.000 | 7.919 | 2036.089 | 7.727 |
| P3 | - | - | - | - | 1.414 |
| EPG treatment* | 0.011 | 0.014 | -0.000(482) | -0.015 | -0.009 |
| KCCQ-CSS: 55.73 to <73.96 (Quartile 2) | -0.317 | -0.328 | 0.325 | 0.263 | 0.221 |
| KCCQ-CSS: 73.96 to <88.02 (Quartile 3) | -0.580 | -0.594 | 0.523 | 0.457 | 0.415 |
| KCCQ-CSS: 88.02 to 100 (Quartile 4) | -0.933 | -0.955 | 0.819 | 0.726 | 0.678 |

EPG = empagliflozin; KCCQ-CSS = Kansas City Cardiomyopathy Questionnaire Clinical Symptom Score

* Set to zero to ensure clinical plausibility consistency with the base case (Weibull distribution)

Table G. Population-specific risk equations for cardiovascular death (Weibull distribution)

|  | Coefficient | | | SE |
| --- | --- | --- | --- | --- |
| Covariate | **ITT population** | **T2D** | **Non-T2D** |  |
| Shape | 0.201 | 0.200 | 0.202 | 0.207 |
| Scale | -10.150 | -10.150 | -10.156 | 0.302 |
| EPG treatment effect | -0.082 | 0.012 | -0.186 | 0.286 |
| Updated KCCQ-CSS: relative to KCCQ-CSS 0 to <55.73 (Quartile 1) | | | | |
| KCCQ-CSS: 55.73 to <73.96 (Quartile 2) | -0.328 | -0.304 | -0.349 | 0.294 |
| KCCQ-CSS: 73.96 to <88.02 (Quartile 3) | -0.709 | -0.736 | -0.672 | 0.320 |
| KCCQ-CSS: 88.02 to 100 (Quartile 4) | -1.147 | -1.103 | -1.176 | 0.335 |

EPG = empagliflozin; KCCQ-CSS = Kansas City Cardiomyopathy Questionnaire Clinical Symptom Score; SE = standard error; T2D = type 2 diabetes

Table H. Risk equations for cardiovascular death based on alternative distributions for ITT

| Covariate | Exponential | Gompertz | Log-Normal | Log-Logistic | Generalised Gamma |
| --- | --- | --- | --- | --- | --- |
| P1 | - | 0.001 | 1.852 | 1.257 | 0.465 |
| P2 (intercept) | 0.000(173) | 0.000(130) | 8.761 | 3622.985 | 8.204 |
| P3 | - | - | - | - | 1.810 |
| EPG treatment | -0.085 | -0.083 | 0.092 | 0.068 | 0.066 |
| KCCQ-CSS: 55.73 to <73.96 (Quartile 2) | -0.318 | -0.326 | 0.377 | 0.287 | 0.248 |
| KCCQ-CSS: 73.96 to <88.02 (Quartile 3) | -0.695 | -0.704 | 0.688 | 0.598 | 0.560 |
| KCCQ-CSS: 88.02 to 100 (Quartile 4) | -1.123 | -1.137 | 1.036 | 0.953 | 0.921 |

EPG = empagliflozin; KCCQ-CSS = Kansas City Cardiomyopathy Questionnaire Clinical Symptom Score

Table I. Risk equation for EPG discontinuation for all populations (Generalised gamma distribution)

| Covariate | Coefficient | SE |
| --- | --- | --- |
| Parameter 1 | 0.650 | 0.659 |
| Parameter 2: Intercept | 7.504 | 0.263 |
| Parameter 3 | 1.846 | 0.234 |
| Updated KCCQ-CSS: ref to KCCQ-CSS 0 to <55.73 (Quartile 1) | | |
| KCCQ-CSS: 55.73 to <73.96 (Quartile 2) | 0.300 | 0.262 |
| KCCQ-CSS: 73.96 to <88.02 (Quartile 3) | 0.427 | 0.264 |
| KCCQ-CSS: 88.02 to 100 (Quartile 4) | 0.757 | 0.273 |

KCCQ-CSS = Kansas City Cardiomyopathy Questionnaire Clinical Symptom Score; SE = standard error

Table J. Risk equations for EPG discontinuation based on alternative distributions

| Covariate | Exponential | Gompertz | Log-Normal | Log-Logistic | Weibull |
| --- | --- | --- | --- | --- | --- |
| P1 | - | 0.000 | 2.030 | 1.000 | -0.087 |
| P2 (intercept) | -7.360 | 0.001 | 7.374 | 1258.810 | -6.816 |
| P3 | - | - | - | - | - |
| KCCQ-CSS: 55.73 to <73.96 (Quartile 2) | -0.336 | -0.335 | 0.429 | 0.400 | -0.327 |
| KCCQ-CSS: 73.96 to <88.02 (Quartile 3) | -0.445 | -0.442 | 0.435 | 0.489 | -0.429 |
| KCCQ-CSS: 88.02 to 100 (Quartile 4) | -0.785 | -0.781 | 0.965 | 0.884 | -0.763 |

Table K. Rates of adverse events

| Adverse event | Event rate per 100 patient-years in EMPEROR-Preserved trial | |
| --- | --- | --- |
|  | **SoC** | **EPG + SoC** |
| Acute renal failure | 7.26 | 6.87 |
| Bone fracture | 2.30 | 2.43 |
| Genital infection | 0.39 | 1.20 |
| Hepatic injury | 2.84 | 2.08 |
| Hypoglycaemic event* | 1.41 | 1.31 |
| Hypotension | 4.80 | 5.88 |
| Urinary tract infection | 4.53 | 5.56 |
| Volume depletion | 5.38 | 6.78 |
| Ketoacidosis** | 0.90 | 0.78 |

EPG: empagliflozin; LVEF: left ventricular ejection fraction; SoC: standard of care

* Defined as an event with a plasma glucose value of ≤3.9 mmol/L or where assistance was required.

** Ketoacidosis was not included in the base case analysis; its impact was explored in a scenario analysis.
