# Supplementary material for: Cost-effectiveness of empagliflozin in the treatment of Malaysian patients with chronic heart failure and preserved or mildly reduced ejection fraction
Source: PLoS One. 2024 Aug 23;19(8):e0305257. doi: 10.1371/journal.pone.0305257 (PMC11343421; doi:10.1371/journal.pone.0305257)
Supplement: S3 File — (DOCX) [file pone.0305257.s003.docx]

# S3 File. Costs

Table L. Inflation table

| Time | CPI health domain | Factor to 2022 |
| --- | --- | --- |
| 2008 | 96.30 | 1.30 |
| 2009 | 98.40 | 1.27 |
| 2010 | 100.00 | 1.25 |
| 2011 | 102.70 | 1.22 |
| 2012 | 104.80 | 1.20 |
| 2013 | 106.80 | 1.17 |
| 2014 | 109.90 | 1.14 |
| 2015 | 114.80 | 1.09 |
| 2016 | 117.90 | 1.06 |
| 2017 | 120.90 | 1.04 |
| 2018 | 121.90 | 1.03 |
| 2019 | 122.70 | 1.02 |
| 2020 | 124.10 | 1.01 |
| 2021 | 124.70 | 1.01 |
| 2022* | 125.40 | 1.00 |

CPI = consumer price index

Source: Consumer Price Index (CPI), Department of Statistics Malaysia (last update: January 2023) (1)

Table M. HF drug class, member drugs, dosage, market share and estimated monthly cost by treatment class (based on Q2/2022 IQVIA drug pricing database).

| Drug class | Drug | Strength | Daily dosage | Daily cost (RM) | Monthly cost (RM)* | Share (within-class)** | Weighted average monthly cost (by class), RM |
| --- | --- | --- | --- | --- | --- | --- | --- |
| SGLT2i | EPG | 10 mg | 10 mg | 3.64 | 110.66 | 100.0% | 110.66 |
| ACEi | Enalapril | 20 mg | 20 mg | 1.20 | 36.44 | 0.7% | 14.54 |
|  | Ramipril | 5 mg | 5 mg | 1.24 | 37.80 | 1.1% |  |
|  | Perindopril | 4 mg | 4 mg | 0.46 | 14.12 | 98.2% |  |
| ARB | Losartan | 50 mg | 50 mg | 2.34 | 71.19 | 35.0% | 95.79 |
|  | Valsartan | 80 mg | 160 mg | 1.79 | 109.03 | 65.0% |  |
| ARNi | Sacubitril / Valsartan | 100 mg | 200 mg | 12.71 | 386.88 | 100.0% | 386.88 |
| BB | Bisoprolol | 5 mg | 5 mg | 1.43 | 43.47 | 86.8% | 43.44 |
|  | Carvedilol | 25 mg | 25 mg | 1.42 | 43.26 | 13.2% |  |
| Ivabradine | Ivabradine | 5 mg | 10 mg | 6.16 | 187.36 | 100.0% | 187.36 |
| Loop diuretics | Frusemide | 40 mg | 40 mg | 0.10 | 3.04 | 95.0% | 6.64 |
|  | Bumetanide | 1 mg | 2 mg | 2.46 | 74.88 | 5.0% |  |
| MRA | Spironolactone | 25 mg | 25 mg | 0.46 | 13.93 | 100.0% | 13.93 |

ACEi = angiotensin-converting enzyme inhibitor; ARB = angiotensin receptor blocker; ARNi = angiotensin receptor neprilysin inhibitor; BB = beta-blocker; EPG = empagliflozin; MRA = mineralocorticoid receptor antagonist; SGLT2i = sodium-glucose cotransporter-2 inhibitor

* Monthly cost of each drug is the product of daily cost and number of days per month (365.25 days/year divided by 12 months/year)

** Market share for each drug was estimated based on local utilisation with confirmation from clinical experts.

Table N. Estimated monthly cost of SoC for all populations (based on Q2/2022 IQVIA drug pricing database).

| Treatment regimen | ITT | | T2D subgroup | | Non-T2D subgroup | |
| --- | --- | --- | --- | --- | --- | --- |
|  | Utilisation at baseline in EMPEROR-Preserved trial | Monthly cost of drug class (RM) | Utilisation at baseline in EMPEROR-Preserved trial | Monthly cost of drug class (RM) | Utilisation at baseline in EMPEROR-Preserved trial | Monthly cost of drug class (RM) |
| SGLT2i (EPG) | For EPG plus SoC arm only: 110.66 | | | | | |
| ACEi | 40.2% | 5.85 | 40.2% | 5.84 | 40.3% | 5.86 |
| ARB | 38.7% | 37.05 | 41.6% | 39.87 | 35.8% | 34.33 |
| ARNi | 2.2% | 8.66 | 2.1% | 8.30 | 2.3% | 9.01 |
| BB | 86.3% | 37.49 | 87.9% | 38.18 | 84.8% | 36.82 |
| Ivabradine | 1.2% | 2.22 | 1.2% | 2.30 | 1.1% | 2.15 |
| Loop diuretics* | 67.7% | 4.49 | 67.7% | 4.49 | 67.7% | 4.49 |
| MRA | 37.5% | 5.22 | 39.1% | 5.44 | 35.9% | 5.01 |
| Weighted average monthly cost of SoC (RM) | 100.98 | | 104.42 | | 97.66 | |
| Weighted average monthly cost of EPG plus SoC (RM) | 100.98 + 110.66  = 211.64 | | 104.42 + 110.66  = 215.08 | | 97.66 + 110.66  = 208.32 | |

ACEi = angiotensin-converting enzyme inhibitor; ARB = angiotensin receptor blocker; ARNi = angiotensin receptor neprilysin inhibitor; BB = beta-blocker; MRA = mineralocorticoid receptor antagonist; SGLT2i = sodium-glucose cotransporter-2 inhibitor

* Utilisation of loop diuretics was assumed equivalent for all populations.

Table O. Types of CV deaths and costs per episode

| Type of CV death | Cost of fatal event in 2008 (RM)* | | Source |
| --- | --- | --- | --- |
|  | Male | Female |  |
| Major coronary | 601 | 619 | Cost calculator by Clarke et al. (2010), based on Malaysian subjects in ADVANCE trial (2) |
| Major cerebrovascular | 3,556 | 3,730 |  |
| Heart failure | 831 | 858 |  |

CV: cardiovascular. Link to Clarke and colleagues’ cost calculator: https://doi.org/10.1371/journal.pmed.1000236.s001

* The cost of a fatal CV event for male patient was determined by the following input settings: country/region: Malaysia; age: 60 (based on mean age of HF patients in Malaysia Heart Failure (MYHF) Registry 2021), male: 1, hospital type: tertiary, and the average cost per event (2008, RM) are displayed under the headings of “Events in this year, fatal”. For female patients, the same input settings were used but the field for male was set to 0.

Table P. Percentages of male and female patients in the ITT population, T2D and non-T2D subpopulations

| Sex | ITT population | T2D subgroup | Non-T2D subgroup | Source |
| --- | --- | --- | --- | --- |
| Male | 55.3% | 57.2% | 53.4% | EMPEROR-Preserved trial |
| Female | 44.7% | 42.8% | 46.6% |  |

HF: heart failure; LVEF: left ventricular ejection fraction; T2D:

Table Q. Gender-weighted cost of fatal CV event and weighted average cost of CV death per event for the ITT population.

| Type of CV death | Number of cases (2020)* | Gender-weighed cost of fatal event for the ITT in 2008 (RM) | Weighted average cost of CV death for the ITT, per event in 2008 (RM) | Weighted average cost of CV death for the ITT, per event in 2022 (RM)** |
| --- | --- | --- | --- | --- |
| Major coronary | 2,668 | 609 | 1,976 | 2,575*** |
| Major cerebrovascular | 2882 | 3,634 |  |  |
| Heart failure | 911 | 843 |  |  |

CV: cardiovascular; HF: heart failure;

*Source: MY-DRG Casemix Database 2020

*Cost has been inflation-adjusted using the health care component of the Consumer Price Index.

*** RM 2,573 for T2D subgroup and 2,577 for non-T2D subgroup

Table R. Estimated per-episode costs of treatment-emergent adverse events

| Adverse event | Cost in 2022  (RM per episode) | | Distribution of visit type* | | Weighted average cost per episode in 2022 (RM) |
| --- | --- | --- | --- | --- | --- |
|  | **Outpatient*** | **Inpatient**** | **Outpatient** | **Inpatient** |  |
| Urinary tract infection | 176 | 213 | 99% | 1% | 213 |
| Genital infection | 271 | 5,203 | 99% | 1% | 320 |
| Acute renal failure | 400 | 3,881 | 20% | 80% | 3,185 |
| Hepatic injury | 440 | 3,928 | 20% | 80% | 3,230 |
| Volume depletion | 400 | 3,322 | 80% | 20% | 984 |
| Hypotension | 320 | 5,129 | 80% | 20% | 1,282 |
| Confirmed hypoglycaemia | 120 | 5,848 | 90% | 10% | 693 |
| Bone fracture | 5,609 | 3,523 | 0% | 100% | 3,523 |
| Ketoacidosis*** | - | 4,705 | 0% | 100% | 4,705 |

* The distribution of visit type and health resource utilisation were estimated based on inputs from clinical experts.

** The inpatient cost of AE was based on 2019 data from Malaysian Diagnosis-Related Group (MY-DRG) Casemix Database, inflated to 2022 values.

** Ketoacidosis was not included in the base case analysis; its impact was explored in a scenario analysis. The inpatient cost of ketoacidosis was estimated from standard treatment algorithm in consultation with clinical experts. Moreover, ketoacidosis was assumed to be manageable only in an inpatient setting.

Table S. Estimated monthly cost of disease management

| Health state | Monthly visit frequency* | Unit cost of HF clinic visit in 2021 (RM)* | Estimated monthly cost of outpatient HF care | |
| --- | --- | --- | --- | --- |
|  |  |  | 2021 (RM) | 2022 (RM)** |
| KCCQ-CSS quartile 1 | 0.2941 | 96.17 | 28.28 | 28.44 |
| KCCQ-CSS quartile 2 | 0.2941 | 96.17 | 28.28 | 28.44 |
| KCCQ-CSS quartile 3 | 0.2941 | 96.17 | 28.28 | 28.44 |
| KCCQ-CSS quartile 4 | 0.2941 | 96.17 | 28.28 | 28.44 |

KCCQ-CSS = Kansas City Cardiomyopathy Questionnaire Clinical Symptom Score; HF = heart failure

* Source: S. C. Ong et al. (2022), with cost of medications excluded to avoid double counting (3)

** Cost has been inflation-adjusted using the health care component of the Consumer Price Index.

**References**

1. Department of Statistics Malaysia. Consumer Price Index, Malaysia, December 2022 [Internet]. Department of Statistics Malaysia; 2022 [cited 2022 Feb 20]. Available from: https://www.dosm.gov.my/v1/index.php?r=column/cthemeByCat&cat=106&bul_id=MWsxc25MWVgyK3JrTjdqU1VRTXpIUT09&menu_id=bThzTHQxN1ZqMVF6a2I4RkZoNDFkQT09

2. Clarke PM, Glasziou P, Patel A, Chalmers J, Woodward M, Harrap SB, et al. Event Rates, Hospital Utilization, and Costs Associated with Major Complications of Diabetes: A Multicountry Comparative Analysis. PLOS Medicine. 2010 Feb 23;7(2):e1000236.

3. Ong SC, Low JZ, Yew WY, Yen CH, Abdul Kader MASK, Liew HB, et al. Cost analysis of chronic heart failure management in Malaysia: A multi-centred retrospective study. Frontiers in Cardiovascular Medicine [Internet]. 2022 [cited 2022 Nov 11];9. Available from: https://www.frontiersin.org/articles/10.3389/fcvm.2022.971592
