# Supplementary material for: Cost-effectiveness of empagliflozin in the treatment of Malaysian patients with chronic heart failure and preserved or mildly reduced ejection fraction
Source: PLoS One. 2024 Aug 23;19(8):e0305257. doi: 10.1371/journal.pone.0305257 (PMC11343421; doi:10.1371/journal.pone.0305257)
Supplement: S4 File — (DOCX) [file pone.0305257.s004.docx]

# S4 File. Utilities

Table T. Health-related quality of life equation derived from EMPEROR-Preserved through a linear mixed model.

| Covariate | Coefficient | SE |
| --- | --- | --- |
| Intercept | 0.605 | 0.06 |
| Baseline EQ-5D (standardised) | 0.088 | 0.002 |
| **eGFR ≥60 (ref: eGFR < 60)** | 0.010 | 0.003 |
| **Age ≥ 65 (ref: Age < 65)** | -0.012 | 0.004 |
| **Male (ref: Female)** | 0.011 | 0.003 |
| **Race (ref: White)** | | |
| Asian | 0.028 | 0.013 |
| Black | -0.005 | 0.008 |
| Multiple | -0.014 | 0.011 |
| Native | 0.016 | 0.009 |
| Pacific | 0.003 | 0.021 |
| Unknown | 0.017 | 0.092 |
| **Region (ref: Europe)** | | |
| Latin America | 0.006 | 0.004 |
| North America | -0.001 | 0.005 |
| Asia | -0.032 | 0.014 |
| Other | 0.009 | 0.008 |
| **Prior atrial fibrillation or flutter (ref: No)** | | |
| Yes | 0.009 | 0.003 |
| Unknown | -0.080 | 0.046 |
| Baseline BMI (standardised) | -0.005 | 0.002 |
| **Treatment-emergent adverse event in prior month (ref: No event ever/in prior month)** | | |
| Hepatic injury | -0.042 | 0.016 |
| Urinary Tract Infection | -0.04 | 0.013 |
| Genital Mycotic Infection | 0.08 | 0.033 |
| Volume depletion | -0.026 | 0.013 |
| Bone fracture | -0.156 | 0.024 |
| Acute renal failure | -0.013 | 0.010 |
| Hypoglycaemic event | 0.015 | 0.021 |
| Ketoacidosis* | 0.017 | 0.027 |
| **hHF Events in prior year (ref: No hHF events ever/in past year)** | | |
| hHF in prior 0-1 month | -0.047 | 0.013 |
| hHF in prior 1-2 months | -0.058 | 0.016 |
| hHF in prior 2-4 months | -0.042 | 0.012 |
| hHF in prior 4-12 months | -0.018 | 0.008 |
| **Updated KCCQ-CSS: ref to KCCQ-CSS 0 to <55.73 (Quartile 1)** | | |
| KCCQ-CSS: 55.73 to <73.96 (Quartile 2) | 0.094 | 0.004 |
| KCCQ-CSS: 73.96 to <88.02 (Quartile 3) | 0.165 | 0.004 |
| KCCQ-CSS: 88.02 to 100 (Quartile 4) | 0.219 | 0.004 |

AE = adverse event; CV = cardiovascular; hHF = hospitalisation due to heart failure; eGFR = estimated glomerular filtration rate; KCCQ-CSS = Kansas City Cardiomyopathy Questionnaire Clinical Symptom Score; SE = standard error

* Ketoacidosis was not included in the base case analysis; its impact was explored in a scenario analysis.

** Disutility due to hHF (over one year) was estimated as a weighted average of the relevant coefficients from the regression model (each coefficient represents a different duration of time elapsed since the event occurred), and their respective duration of time elapsed since the event occurred: hHF dis-utility = -0.047*1 + (-0.058*1) + (-0.042*2) + (-0.018*8) = -0.335.
