# Supplementary material for: Cost-effectiveness of empagliflozin in the treatment of Malaysian patients with chronic heart failure and preserved or mildly reduced ejection fraction
Source: PLoS One. 2024 Aug 23;19(8):e0305257. doi: 10.1371/journal.pone.0305257 (PMC11343421; doi:10.1371/journal.pone.0305257)
Supplement: S5 File — (DOCX) [file pone.0305257.s005.docx]

# S5 File. Sensitivity analyses

Table U. Parameters varied and distributions used in probabilistic sensitivity analysis

| Parameter | Probabilistic distribution used to vary model parameters |
| --- | --- |
| Coefficients of the equations used to estimate the rates of hHF, all-cause death, CV death, time to treatment discontinuation and health state utility | Multivariate normal distributions using the lower-triangular matrices obtained from Cholesky decomposition of covariance matrices |
| KCCQ-CSS health state transition probabilities | Dirichlet distributions |
| Adverse event rates and costs of clinical events, disease management, and adverse event management | Gamma distributions. Parameters draws utilised standard error within 20% of the mean values. |
| Adverse event dis-utilities | Beta distribution |

CV = cardiovascular; KCCQ-CSS = Kansas City Cardiomyopathy Questionnaire Clinical Symptom Score; hHF = hospitalisation for heart failure

Table V. Base-case analysis and PSA results for HFrEF (starting age: 60 years old)

|  | Deterministic | | | Probabilistic (mean and 95% CI) | | |
| --- | --- | --- | --- | --- | --- | --- |
| Outcome | EPG + SoC | SoC | Incremental | EPG + SoC | SoC | Incremental |
| Total cost | RM25,410 | RM21,758 | RM3,652 | RM24,160  (15,574 to 35,568) | RM21,429 (14,556 to 30,316) | RM2731  (31 to 8,454) |
| Total QALYs | 3.64 | 3.46 | 0.18 | 3.58  (2.87 to 4.23) | 3.46  (2.83 to 4.08) | 0.12  (-0.48 to 0.98) |
| **ICER, cost per QALY gained** | RM20,364 | | | Mean: RM 22,134 | | |

CI = confidence interval; EPG = empagliflozin; ICER = incremental cost-effectiveness ratio; LY = life years; QALYs = quality-adjusted life years; SoC = standard of care
