# Supplementary material for: Cost-effectiveness of empagliflozin in the treatment of Malaysian patients with chronic heart failure and preserved or mildly reduced ejection fraction
Source: PLoS One. 2024 Aug 23;19(8):e0305257. doi: 10.1371/journal.pone.0305257 (PMC11343421; doi:10.1371/journal.pone.0305257)
Supplement: S6 File — (DOCX) [file pone.0305257.s006.docx]

# S6 File. Results

Table W. Deterministic sensitivity analyses inputs and results (ITT population)

| Scenario | Base Case Input | Alternative Input | Description | ICER per QALY | % change from base-case ICER |
| --- | --- | --- | --- | --- | --- |
| **Clinical Inputs** | | | | | |
| CV & all-cause death: Distribution | Weibull | Exponential | Alternative distribution | RM41,111 | +1.6% |
| CV death: Treatment effect of EPG | -0.0818 | 0 | Removing treatment effect of EPG | RM58,070 | +43.5% |
| Non-CV death: adjust with Malaysia’s lifetable? | Yes | No | Using non-CV death rates predicted from risk equation | RM41,626 | +2.9% |
| hHF: Treatment effect | -0.254 | -0.186 | Upper confidence interval limit (0.83) | RM53,733 | +32.8% |
|  |  | -0.511 | Lower confidence interval limit (0.60) | RM 33,118 | -18.1% |
| Include EPG discontinuation? | Yes | No | Alternative scenario for discontinuation | RM41,084 | +1.6% |
| Discontinuation: Distribution | Generalised Gamma | Weibull | Alternative distribution | RM43,083 | +6.5% |
| **Costs and Resource Use** | | | | | |
| Cost of hHF | RM5,276 | RM4,221 | Decrease by 20% | RM41,109 | +1.6% |
|  |  | RM6,332 | Increase by 20% | RM39,799 | -1.6% |
| Cost of CV death | RM2,573 | RM2,058 | Decrease by 20% | RM40,519 | +0.2% |
|  |  | RM3,087 | Increase by 20% | RM40,388 | -0.2% |
| Unit Costs of Disease Management | RM97 | RM77 | Decrease by 20% | RM40,403 | -0.1% |
|  |  | RM116 | Increase by 20% | RM40,504 | +0.1% |
| Monthly Cost of Disease Management: KCCQ-CSS 1st Quartile | RM28 | RM23 | Decrease by 20% | RM40,498 | +0.1% |
|  |  | RM34 | Increase by 20% | RM40,410 | -0.1% |
| Monthly Cost of Disease Management: KCCQ-CSS 2nd Quartile | RM28 | RM23 | Decrease by 20% | RM40,491 | +0.1% |
|  |  | RM34 | Increase by 20% | RM40,417 | -0.1% |
| Monthly Cost of Disease Management: KCCQ-CSS 3rd Quartile | RM28 | RM23 | Decrease by 20% | RM40,420 | -0.1% |
|  |  | RM34 | Increase by 20% | RM40,488 | +0.1% |
| Monthly Cost of Disease Management: KCCQ-CSS 4th Quartile | RM28 | RM23 | Decrease by 20% | RM40,357 | -0.2% |
|  |  | RM34 | Increase by 20% | RM40,551 | +0.2% |
| Cost of AE management | Multiple Values | Multiple Values | Decrease by 20% | RM40,379 | -0.2% |
|  |  |  | Increase by 20% | RM40,529 | +0.2% |
| Empagliflozin cost per day | RM 1.69 | RM1.35 | Decrease by 20% | RM31,947 | -21.0% |
|  |  | RM2.03 | Increase by 20% | RM48,961 | +21.0% |
| **Utilities** | | | | | |
| Utility: KCCQ-CSS 1st Quartile | 0.6131 | 0.6058 | Lower 95% CI | RM40,551 | +0.2% |
|  |  | 0.6204 | Upper 95% CI | RM40,644 | +0.5% |
| Utility: KCCQ-CSS 2nd Quartile | 0.7071 | 0.6998 | Lower 95% CI | RM40,294 | -0.4% |
|  |  | 0.7144 | Upper 95% CI | RM40,614 | +0.4% |
| Utility: KCCQ-CSS 3rd Quartile | 0.7777 | 0.7697 | Lower 95% CI | RM40,614 | +0.4% |
|  |  | 0.7855 | Upper 95% CI | RM40,296 | -0.4% |
| Utility: KCCQ-CSS 4th Quartile | 0.8319 | 0.8235 | Lower 95% CI | RM40,947 | +1.2% |
|  |  | 0.8403 | Upper 95% CI | RM39,981 | -1.2% |
| Disutility: hHF | -0.3354 | -0.2611 | Lower 95% CI | RM44,201 | +9.3% |
|  |  | -0.4097 | Upper 95% CI | RM36,958 | -8.6% |
| Disutility: AEs | Multiple Values | Multiple Values | Lower 95% CI | RM40,366 | -0.2% |
|  |  |  | Upper 95% CI | RM40,499 | +0.1% |

AE = adverse event; CI = confidence interval; CV = cardiovascular; EPG = empagliflozin; hHF = hospitalisation for heart failure; ICER = incremental cost-effectiveness ratio; KCCQ-CSS = Kansas City Cardiomyopathy Questionnaire Clinical Symptom Score; QALY = quality-adjusted life year
